# Transitional analysis for multi-objective operative improvement of reformate quality and hydrogen production from a naphtha catalytic reforming process

**DOI:** 10.1016/j.heliyon.2024.e41428

**Published:** 2024-12-21

**Authors:** F. Velázquez-Alonso, C.A. González-Ramírez, J.R. Villagómez-Ibarra, E.M. Otazo-Sánchez, M. Hernández-Juárez, F. Pérez-Villaseñor, A. Castro-Agüero, L.O. Alemán-Vázquez

**Affiliations:** a Área Académica de Química, Instituto de Ciencias Básicas e Ingeniería, Universidad Autónoma del Estado de Hidalgo. Ciudad del Conocimiento, Carretera Pachuca-Tulancingo Km. 4.5, C.P. 42184, Mineral de la Reforma, Hidalgo, Mexico; b Departamento de Ingeniería Química y Bioquímica, Tecnológico Nacional de México campus Pachuca, Carretera México-Pachuca, km 87.5, Col. Venta Prieta, C.P. 42080, Pachuca de Soto, Hidalgo, Mexico; c Facultad de Ciencias Básicas, Ingeniería y Tecnología, Universidad Autónoma de Tlaxcala, Carretera Apizaquito S/N, San Luis Apizaquito, C.P. 90401, Apizaco, Tlaxcala, Mexico; d Instituto Mexicano del Petróleo, Eje Central Lázaro Cárdenas Norte 152, Col. San Bartolo Atepehuacán, C.P. 07730, Ciudad de México, Mexico

**Keywords:** Catalytic naphtha reforming, Hydrogen recovery, Operative transition, Multi-objective optimization

## Abstract

The hydrogen produced (*H*_*2*_) in the Catalytic Naphtha Reforming (CNR) is important in quantity and quality, for the feedback of the process and for supplying the hydrotreatment processes in current refineries. In this work it is presented a study by process simulation using *Aspen HYSYS*® for finding operative transitional modes that simultaneously improve quality of the reformate and hydrogen production of the CNR. The operative conditions that were studied correspond to the recirculation ratio of hydrogen/hydrocarbon (*H*_*2*_*/HC*), with values between 2 and 6, and the temperature (*T*), between 450 and 525 °C, in order to determining the best operative transitional route from the initial operative state to a local improved state, applying the method of superposition of response surfaces and criteria assessment of improvement in quality and quantity of hydrogen produced. A numerical multi-objective operative improvement analysis was performed resulting the objective variables as: Research Octane Number (RON) = 90.72, mass fraction of *H*_*2*_ produced (*%m of H*_*2*_) = 2.9, quality of recycled *H*_*2*_ (*yH*_*2*_)_*R*_ = 0.87, and quality of produced hydrogen (*yH*_*2*_)_*S*_ = 0.9653. Experimental pilot plant data and full-scale industrial data were compared with simulations observing significant similitudes.

## Graphical abstract

Schematic Transitional Operative Routes (TORs) on a multivariate surface response of the industrial, optimum simulated, and pilot plant operative states for: a) Research Octane number (*RON*) and b) molar fraction of recirculated hydrogen (*yH*_*2*_)_*R*_.

**Figure:**
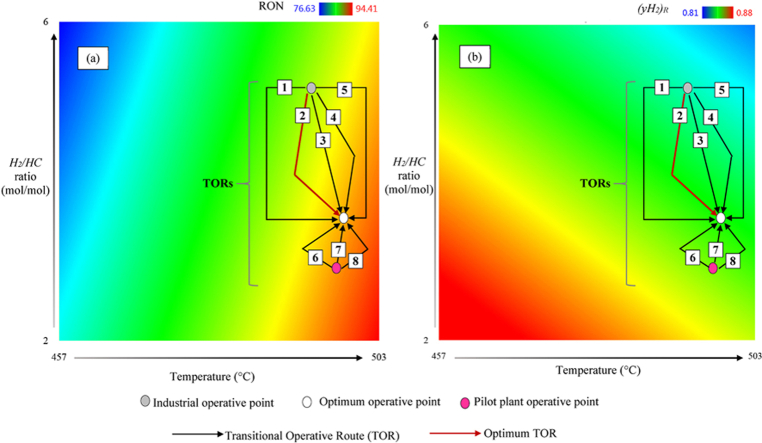

## 1 Introduction

Oil refining industry plays an important role, due that most of its products are part of the energy market generating an impact on world economy. One of the main objectives of this industry is fuel production from crude oil transformation. The Catalytic Naphtha Reforming (CNR) process is one of the most important in refineries, since it consists in the treatment of naphtha (a mixture of hydrocarbons with low octane number), in presence of a dual catalyst Pt-Re/Al_2_O_3_ for obtaining higher octane gasoline, with a higher concentration of aromatic compounds [1,2]. Hydrogen is considered an important byproduct from catalytic reforming, which is useful for downstream hydrotreatment processes within refineries. For this reason, there is a need of developing studies focusing process conditions for improving operational approaches, aiming to increase yield [3]. However, it is necessary to analyze hydrogen production processes focusing its recovery and use as a clean fuel that could provide energetic incentives along with minimization of the environmental impact of fuels [4]. Previous research [5] has been focused on analyzing hydrogen production from catalytic reforming, as a method of obtaining sustainable energy, by considering environmental and economic aspects on a hydrogen-based energy system, concluding that there exist significant effects on lowering Greenhouse Gas Emissions (GGE) during production, fabrication and implementation stages of such system.

Hydrogen production depends upon dehydrogenation reactions in which aromatic compounds and olefins are formed, additionally to cycling reactions that form naphthenic compounds, which are furtherly integrated into aromatic reactions. Due to the large number of organic reactions and chemical compounds involved in catalytic reforming, operative conditions and viability state of the catalyst being used, play an important role in continuous improvement of the process with the aim of producing high quality hydrogen in enough quantity for sustaining productivity of both, the CNR process itself and further hydrotreatment downstream processes in the refinery. Nevertheless, within the separation process of the reformate there exist operative conditions that directly drive hydrogen recovery and its feedback to the CNR process or re-input as a stream process with added value [6]. The fastest chemical reactions, such as dehydrogenation, can reach thermodynamic equilibrium, whereas the remaining reactions are driven by their own kinetics, thus the increment of reaction temperature along with a pressure reduction, will have a positive effect on thermodynamic viability of naphtha dehydrogenation [7]. Besides, there is a diversity of several chemical reactions during catalytic reforming, among which naphtha dehydrogenation and paraffins aromatization can produce hydrogen, whilst n-paraffins hydrocracking is an exothermic reaction that consumes hydrogen [8]. For these reasons, it results significant to perform an operative transitional analysis during the continuous improvement of the CNR process, keeping hydrogen production as an objective.

The CNR process has as main variables: reactor pressure, reaction temperature, space velocity, and the molar feed ratio of hydrogen-hydrocarbons [9]. Hydrogen is essential for avoiding catalyst deactivation and undesirable secondary reactions. Former process configurations used *H*_*2*_*/HC* molar ratios between 8 and 10, whereas modern reformers using highly active catalysts, can operate in a range from 2 to 5 [10]. Since hydrogen is one of the most important compounds in material streams for the modern chemical industry, it shows processing options that allows us to identify operational arrays or models that request an increment of its quality and quantity produced out of catalytic reforming, in an energy market with potentially extended uses of hydrogen. In this way, it has been illustrated the importance of increasing quality and quantity of hydrogen produced from CNR. In this sense, it has being established the importance of analyzing by simulation, several changes in the operative state of CNR, in order to continuously improving quality and quantity of hydrogen production, by setting the Improved Operative Conditions (IOC), along with a transitional analysis for finding different routes towards a change in operative conditions that could result in a better yield and profitability of the process, as well as to maintain a high standard in the Research Octane Number (RON) of the reformate.

### 1.1 Modeling studies about CNR

There also exist studies about new kinetic models, process simulation, and optimization, such as the application of mathematical models for process optimization assuming an unstable operation and feedstock changes, finding a maximum RON value of 94.5, and a maximum hydrogen mass production of 2.15 % [11]. Industrial scale monitoring and process optimization have also been of recent interest [12] when trying to find optimal values of the main operative process variables, through implementation of a historical process data analysis method for operative optimization of a naphtha catalytic reformer, resulting an average temperature of 525 °C in the reaction section.

A data-based model was used for predicting RON values, produced benzene %v/V composition and Reid vapor pressure, in order to determine gasoline quality. This study was carried out in a pilot scale packed bed reactor using a commercial Pt/Al_2_O_3_ catalyst, through manipulation of operative variables like pressure, temperature, space velocity, and *H*_*2*_*/HC* molar ratio. The model considered benzene feedstock composition and isomerization reactions of light naphtha, obtaining correlation coefficients from 0.90 to 0.959 for the results comparison between the proposed model and pilot plant data [13].

Lastly, a recent study was based on development of kinetic expressions at molecular level for generating a mathematical model of the CNR process, which then was compared with experimental runs in a fixed-bed 200 mL reactor within a temperature range from 465 to 490 °C [14].

It is important to highlight that above mentioned investigations have not yet studied a relationship between a technical analysis of the process and alternatives for operative excellence towards continuous improvement that can led to a maximum yield of byproducts (i.e. hydrogen) whilst this work also helps to the establishment of novel optimization methodologies that can be compared at pilot plant and industrial scale. For that reason, this study focuses on a transitional operative analysis from an Initial Operative State (IOS) to an Optimum Operative State (OOS) while keeping a multi-objective approach by simultaneously improving quality and quantity of produced and recycled hydrogen, along with the RON value of the reformate.

### 1.2 Type of process and operative conditions

Technological development of CNR processes started since 1940s when Vladimir Hansel created the first naphtha reforming process in presence of a Pt catalyst. Until 1949 this process was scaled up to the industry, for obtaining high octane gasoline under direction of Universal Oil Products (UOP) company, who named the process as Platforming [15].

Several configurations of the process have been developed on basis of the type of catalyst and the regeneration frequency, therefore, it is possible to find arrangements, such as: Semi-Regenerative (SR) process, Cyclic Regeneration (CR) process and Continuous Catalyst Regeneration (CCR) process [16]. In this work the CCR process was simulated, and then results were compared with industrial CCR data and pilot plant SR data.

#### 1.2.1 Continuous regeneration of catalyst (CCR) process

**Fig. 1:**
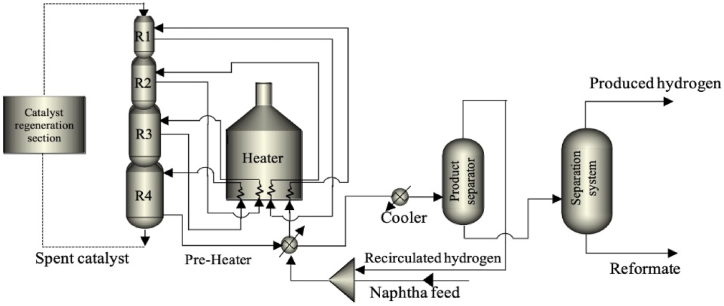
Process flow diagram of a Naphtha Catalytic Reforming process (redrawn from Ref. [18]).

The CCR process (see Fig. 1) is considered one of the most modern methods for naphtha reforming, it consists of 3–4 extended bed reactors connected one on top of each other, in which take place all catalytic reactions, as well as the catalyst is transferred towards an external reactor for coke removal and catalyst regeneration. One of the main advantages of this process is related to the quality of the reformate, since its configuration allows operating with low quality naphtha, in order to produce reformed naphtha with a high RON between 95 and 108 [17], this configuration will also work at low pressure generating a higher hydrogen yield [18].

#### 1.2.2 Process variables and operational conditions

As in any chemical process, manipulation of operative variables directly drives process yield. In case of the CNR process, temperature (*T*), pressure (*P*), and hydrogen/hydrocarbons feed ratio (*H*_*2*_*/HC*), as well as the quality of naphtha fed to the reformer, are some of the variables that affect reaction rate, catalyst activity and, as a consequence, quality of the produced reformate, also hydrogen production can be increased when operating with higher temperature [19]. Theoretical values for the main operative variables are to be found in the following ranges: 450–520 °C, 10–35 bar, and between 3 and 8 mol/mol of the *H*_*2*_*/HC* ratio [20].

Additionally, to the most representative process variables, it is possible to consider characteristics of the feedstock and its effects on specific yield of different chemical species in the reformate, in this sense Honeywell UOP ® has developed an expression to relate general properties in the feedstock, such like API degrees, with naphtha composition in terms of paraffins, naphthenes, and aromatic compounds; which is useful for determining the final contribution of each chemical group to the outcoming yield from the reformer [21].

## 2 Materials and methods

### 2.1 Simulation design

**Fig. 2:**
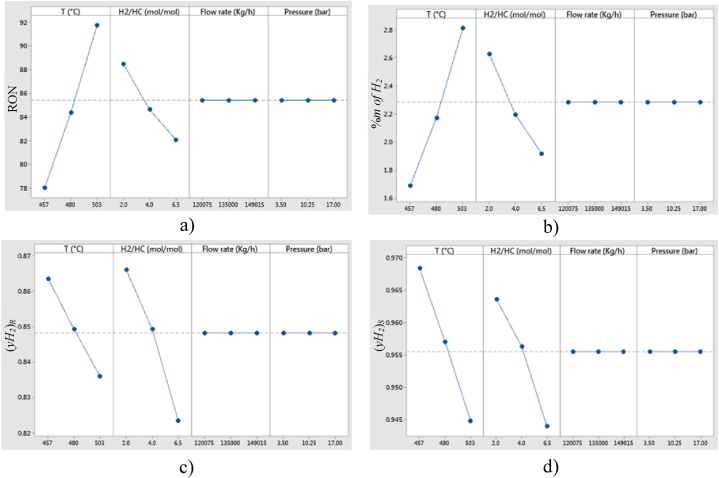
Main effects plots from independent variables on response variables: a) Research Octane Number (RON), b) mass percentage of produced hydrogen (*%m of H*_*2*_), c) molar fraction of recirculated hydrogen *(yH*_*2*_*)*_*R*_, and d) molar fraction of produced hydrogen *(yH*_*2*_*)*_*S*_.

In order to select the most influencing independent variables on response variables, a sensitivity analysis was performed comparing main effects caused by each independent variable: Temperature (*T*), Hydrogen/Hydrocarbon molar feed ratio (*H*_*2*_*/HC*), Feed flowrate (*F*), and Pressure (*P*); on response variables, such as: Research Octane Number (*RON*), mass percentage of produced hydrogen (*%m of H*_*2*_), molar fraction of recirculated hydrogen *(yH*_*2*_*)*_*R*_, and molar fraction of produced hydrogen *(yH*_*2*_*)*_*S*_. Fig. 2(a - d) show the main effects trends that resulted from the sensitivity analysis.

As a result of the sensitivity analysis only independent variables: Temperature (*T*), and Hydrogen/Hydrocarbon molar ratio (*H*_*2*_*/HC*) were selected for further simulations and optimization, since these were the ones that exhibited the main influence on response variables, whereas feed flowrate (*F*) and pressure (*P*) were discarded since these did not cause significant changes on response variables as it can be seen in Fig. 2(a–d).

In this work, operative conditions were taken from industrial scale data, being a feed flowrate of 120,075 kg/h, a reaction temperature between 457 and 503 °C, and an initial RON of 75.25 of the fed naphtha, which comprised a vol/vol composition of 24.78 % of naphthenes, 39.7 % of iso-paraffins, 22.28 % of n-paraffins, 0.34 % of olefins and 12.91 % of aromatic compounds; whereas for the separation system of the reformate and hydrogen produced, a 37.8 °C operative temperature and pressure of 19.3 kg/cm^2^ were considered. Furthermore, an experimental design was defined for simulating the process using *ASPEN HYSYS* ® V8.8 with license number (34.0.0.8909), within its *REFSYS* ® module, considering the identified independent variables for constructing the simulation scenarios as it is reported in Table 1.

**Table 1:** Independent variables and value levels for experimental design of process simulation.

Independent variable	Units	Value levels		
		Low	Medium	High
Temperature (*T*)	°C	457	480	503
*H*_*2*_*/HC* ratio (*H*)	mol/mol	2	4	6

### 2.2 Surface response method (SRM)

The SRM is an experiment design-based strategy and a statistical method for modeling while seeking for improved operational conditions of a process. This is generally applied as an initial experimental stage for determining operability zones or regions when looking for established objectives [22].

Assessment through SMR was based upon a multivariable regression analysis that allowed to determine a relationship between process variables, such as Temperature (*T*) and hydrogen/hydrocarbon molar feed ratio (*H*_*2*_*/HC*), with response variables [23]. Results from process simulations were used for obtaining multivariable statistical models for each one of the response variables that were identified, as shown in Table 2.

**Table 2:** Response variables from the Naphtha Catalytic Reforming process.

Response variable definition	Symbol	Units
Octane number	*RON*	Dimensionless
Mass percent of produced hydrogen	*(%m of H*_*2*_*)*	% (m/m)
Molar fraction of recycled hydrogen (generated from the first separator)	(*yH*_*2*_*)*_*R*_	Mol fraction
Molar fraction of produced hydrogen (generated from the final separation system)	(*yH*_*2*_*)*_*S*_	Mol fraction

Models used in the SRM are polynomic equations that are useful for predicting responses from inlet factors [24], therefore, if there are “*k*” independent variables, a first-degree model has the form of equation (1) [22].

(1) $$Y_{i}=\beta_{0}+\sum_{i=1}^{k}\beta_{i}x_{i}+\varepsilon$$

A second-degree model has the form shown in equation (2)

(2) $$Y_{i}=\beta_{0}+\sum_{i=1}^{k}\beta_{i}x_{i}+\sum_{i=1}^{k}\beta_{ii}x_{i}^{2}+\sum_{i=1}^{k}\sum_{\langle j=1}^{k}\beta_{ij}x_{i}x_{j}+\varepsilon$$

Where $Y_{i}$ is the response variable “*i*”, $\beta_{0}$ is the independent coefficient, also known as the average of responses, and $\beta_{i},{\;\beta }_{ii}$ and $\beta_{ij}$ are coefficients from the statistical model, *x*_*i*_ are the independent variables, and *ε* is the statistic error.

Generated models, for each one of the objective variables, were useful for predicting results from different operative conditions, which were used for obtaining response surfaces, where improved quality characteristics of products are identified, in order to estimate Transitional Operative Routes (TORs) that can be followed towards better operative conditions during the search for improved values of the response variables.

### 2.3 Procedure for identifying TORs

A feasible procedure, for estimating statistical models that allowed the assessment of different TORs, consists of the definition of some sets of operative conditions for simulating the CCR process of the CNR, then by generating results from these simulations it was possible to estimate multivariable surface response models, which once obtained, the alternative transitional routes were analyzed on the surface generated, in order to identifying the most efficient transitional routes between an Initial Operative State (IOS) and an Objective Operative State (OOS) for improving quality and quantity of the reformate and hydrogen produced. This procedure was also performed for identifying operative zones where it was reasonable to analyze the operative impact within the process, emphasizing that, the higher the concentration of hydrogen produced, the higher becomes the profitability of an oil refining system.

### 2.4 Multi-objective improvement

A viable method for multi-objective improvement (local optimization) is a 3 stages procedure, which can initiate with formulation of the optimization problem for determining each objective function, identifying those functions to be minimized or maximized, in order to set the best algorithm for optimization [25]. Established restrictions were applied for delimitating regions, for finding feasible solutions (which fulfill all equations and restrictions) and for non-feasible solutions (which did not fulfill at least one restriction) [26].

By using the SRM and the generated response surfaces, along with contour plots for each one of the response variables, as a function of operative conditions that have been set in this work, it was possible to proceed applying a contour superposition method for delimitating regions where response variables can take feasible and improved values.

### 2.5 Pilot plant experiments

Pilot plant experiments were performed in a semi-regenerative (SR) CNR process, since CCR process is not feasible at pilot plant scale due to the need of using a continuous catalyst circulation and regeneration system, with a riser element for catalyst feedback to the reactor, such a system should be of industrial scale dimensions accordingly to the particle size of the catalyst, in order to avoid attrition effects on the catalyst, that would affect its mechanical resistance and would prevent the possibility to assess its chemical performance. The pilot plant comprises a series of 3 reactors in which the following variables can be changed in the ranges that are described: pressure between 1000 and 190,000 kPa, temperature between 350 and 550 °C and space-velocity between 0.5 and 8 h^−1^. The procedure for carrying out an operative run in the SR-CNR pilot plant is the following.

1. Previous conditioning: this stage consists of reactors packing, filters cleaning, as well as feed tank filling.
2. Leaking tests for hydrogen and nitrogen.
3. Catalyst conditioning.
4. Setting up of the pilot plant and establishment of operative conditions for starting a run.
5. Feeding hydro-desulfurated Naphtha through the system.
6. Checking up of the mass balance.
7. Operation, monitoring and sampling the lines of reformation products and by-products.

Given that the operation is carried out under isothermal conditions, it is suggested to place the catalyst in the middle of each reactor, this is achieved by filling with catalyst the middle part of the reactor adding this in between two bed sides filled with an inert material. At the top, each reactor is filled with inert materials such as, alumina or silicon carbide aiming to preheating or vaporizing the load. In the central zone of each reactor the catalyst is added in a specific volume for testing during reformation. The bottom zone of each reactor is also filled with inert materials like alumina or silicon carbide to avoid heat losses and drops in temperature. The whole volume distributed in each reactor is as follows: 196 cm^3^ for the first reactor, 302 cm^3^ for the second reactor, and 391 cm^3^ for the third reactor; this volume distribution improves flow patterns and increase fluid retention and residence time distribution, while packed inert materials also contribute to reach isothermal conditions.

### 2.6 Analytical methods for products and byproducts quantification

**Fig. 3:**
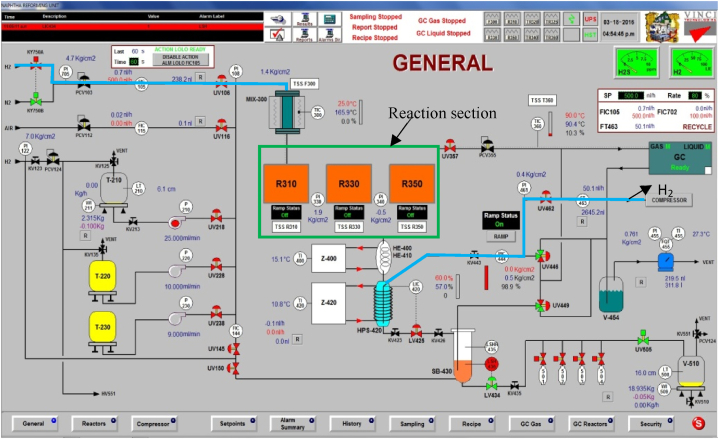
General pilot plant operation panel.

For analyzing and quantifying products and byproducts from naphtha reforming, the pilot plant system is complemented with two on-line gas chromatographers. A general scheme of the experimental system, with the pilot plant, sampling ports, and gas chromatographers, for on -line analysis; is shown in Fig. 3.

#### 2.6.1 Detailed hydrocarbons Analyzer (DHA) by gas Chromatography (GC)

With the aim of analyzing quality and composition of intermediate products that are generated in between reactors, it has been installed an on-line DHA gas chromatographer GC7890B DHA model of the brands Petroleum Analytical Company ®/Analytical Controls ®/Agilent Technologies ®, which includes an automatic sampler for liquid injections, a 5 μL syringe, a capillary column packed with dimethyl-silicone, and a Flame Ionization Detector (FID), for quantifying individual compounds from a hydrocarbons sample, fulfilling D6729, D5134, D6730, and D6733 ASTM methods. This equipment has also an on-line injection system connected to different ports distributed across the pilot plant.

This testing method is useful for identifying individual hydrocarbon compounds from spark ignition engine fuels and fuel mixtures containing mixed oxygenated compounds with boiling point ranges up to 225 °C. It is possible to compare results from this method with other testing methods for certain selected compounds, including olefins and several group types for interlaboratory comparisons. Although, benzene, toluene, and several oxygenated compounds can be determined with DHA-GC, it is possible to obtain a confirmatory analysis using specific testing methods. By following the same operative indications as for a multiple applications GC system, it was established a run analysis for a virgin naphtha sample using the D6729 ASTM method with argon as a carrier gas [27].

By using D6729 ASTM method for DHA-GC systems, it was possible to obtain naphtha composition as a function of different chemical groups and carbon numbers of several compounds, mainly from paraffins, olefins, naphthenes, and aromatics, which allowed a chemical characterization of the type of obtained naphtha along with its physicochemical properties.

#### 2.6.2 Gas Chromatography for determining hydrogen quality

At the beginning of the process, fresh hydrogen was fed to the pilot plant for initiating chemical reactions, if the run continues, fresh hydrogen is gradually replaced by recirculated hydrogen produced from the catalyst, this is done by using an external hydrogen compressor coupled to the pilot plant. This operative mode can simulate at pilot plant scale, real performance of an industrial SR reforming plant. For this reason, it is important to determine quality of recycled hydrogen, which was done by using a gas chromatographer HiSpeed ® model GC 7890B HiSpeed ® of the brands Petroleum Analytical Company ®/Analytical Controls ®/Agilent Technologies ®, with a Thermal Conductivity Detector (TCD), for determining hydrogen molar composition, which is an added value byproduct that is continuously reutilized in the process.

#### 2.6.3 Multidimensional GC

A gas chromatographer model Reformulyzer ® M4 of the brands Petroleum Analytical Company ®/Analytical Controls ®/Agilent Technologies ®, which uses several coupled GC columns with a configuration described in the D6839 ASTM method (Standard Test method for Hydrocarbon Types, Oxygenated Compounds, Benzene, and Toluene in Spark Ignition Fuels by Multidimensional Gas Chromatography) using capillary and micro-packed columns, as well as olefins traps, reducing time analysis and improving analytical precision [28].

### 2.7 Research Octane Number (RON) measurements

RON was estimated by using the DHA-GC method for on-line sampling from the pilot plant, as well as for samples from the feed and reformate product. For RON estimation, GC results are divided into 31 chemical groups, assigning a RON value for each group [29], the RON average value is calculated from equation (3):

(3) $$RON=\sum_{i=1}^{31}W_{i}{RON}_{group}$$

Where *“W*_*i*_*”* is the mass fraction of group *“i”* and *“RON*_*group*_*”* is the octane number assigned to each hydrocarbons group.

During process operation, it is essential to evaluate quality of the reformate. Currently, main chromatographic techniques, for this purpose, are Multidimensional GC and DHA-GC.

## 3 Results and discussion

Using data obtained from simulation, it was built a quadratic multivariate surface response model with interactions between independent variables like temperature (*T*) and the Hydrogen/Hydrocarbon ratio (*H*_*2*_*/HC*), as described in equation (4).

(4) $$y_{i}=a_{0}+a_{1}x_{1}+a_{2}x_{2}+a_{3}x_{1}^{2}+a_{4}x_{2}^{2}+a_{5}x_{1}x_{2}$$

**Fig. 4:**
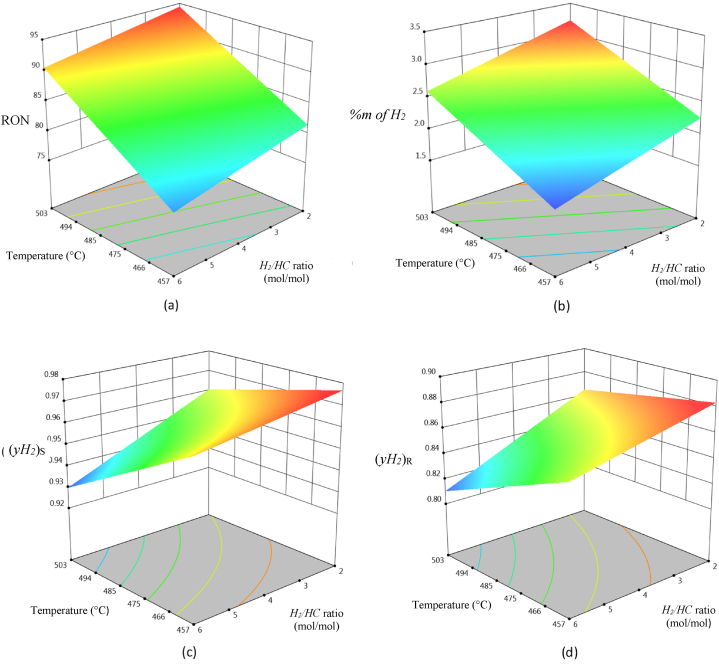
Response surfaces of: (a) octane number (*RON*), (b) mass percentage of produced hydrogen (*% m of H*_*2*_), (c) molar fraction of produced hydrogen leaving the separation system (*yH*_*2*_)_*S*_ and (d) molar fraction of recirculated hydrogen (*yH*_*2*_)_*R*_.

For each dependent variable “*y*_*i*_”, where “*x*_*1*_” is the temperature (*T*) and “*x*_*2*_” is the Hydrogen/Hydrocarbon ratio (*H*_*2*_*/HC*), it was obtained a set of coefficients for each multivariable equation (see Table 3), along with response surface plots shown in Fig. 4(a–d).

**Table 3:** Regression parameters of quadratic response surface models with variables interaction and correlation coefficients of each equation.

y_i_	a_0_	a_1_	a_2_	a_3_	a_4_	a_5_	r^2^
*RON*	59.906	−0.1559	−2.2783	4.52E-4	0.0234	0.0021	**0.9758**
*(%m of H*_*2*_*)*	7.9386	−0.0459	0.0045	7.30E-5	0.0770	4.08E-4	**0.8131**
(*yH*_*2*_*)*_*R*_	−0.5056	0.0059	0.0706	−6.20E-6	−1.34E-4	−1.66E-4	**0.9575**
(*yH*_*2*_*)*_*S*_	−0.0302	0.0043	0.0501	−4.60E-6	−3.34E-5	−1.14E-4	**0.9812**

### 3.1 Operative zones

By using the above described statistical models and data generated from response surfaces, it was possible to identify operative zones for each dependent variable, selecting boundary values for each variable, which allowed to establish the following constraints for a productive quality.

- RON above 87.
- Hydrogen mass fraction in the outlet stream: greater than 0.025.
- Hydrogen purity in the outlet stream from the high-pressure separator above 0.86.
- Hydrogen purity in the outlet stream from the low-pressure separator above 0.95.

**Fig. 5:**
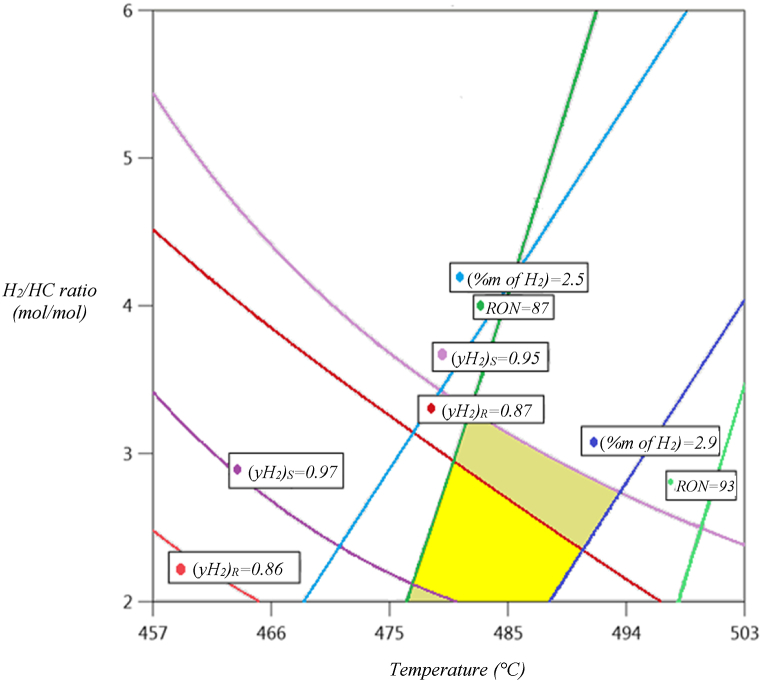
Diagram of operative zones for each dependent variable, remarking the “searching zone” in bright yellow color.

Considering these criteria, operative zones were searched for each dependent variable, observing stripes of acceptable productive quality, as a function of each established constraint. The intersective zone arising from the operative zone for each dependent variable was named as “searching zone” for improved operative conditions that could simultaneously satisfy productive quality criteria as defined (see Fig. 5)

Fig. 5 shows a superposition of operative zones for each dependent variable, giving as a result a “searching zone” that can be found between 479 and 496 °C, and a “*H*_*2*_*/HC*” ratio between 2 and 3.5 mol/mol, therefore it is proposed as an Objective Operative State (OOS) a temperature of 496 °C, and a “*H*_*2*_*/HC*” ratio of 2, starting from and Initial Operative State (IOS) with a temperature of 450 °C and a “*H*_*2*_*/HC*” ratio of 6.

**Fig. 6:**
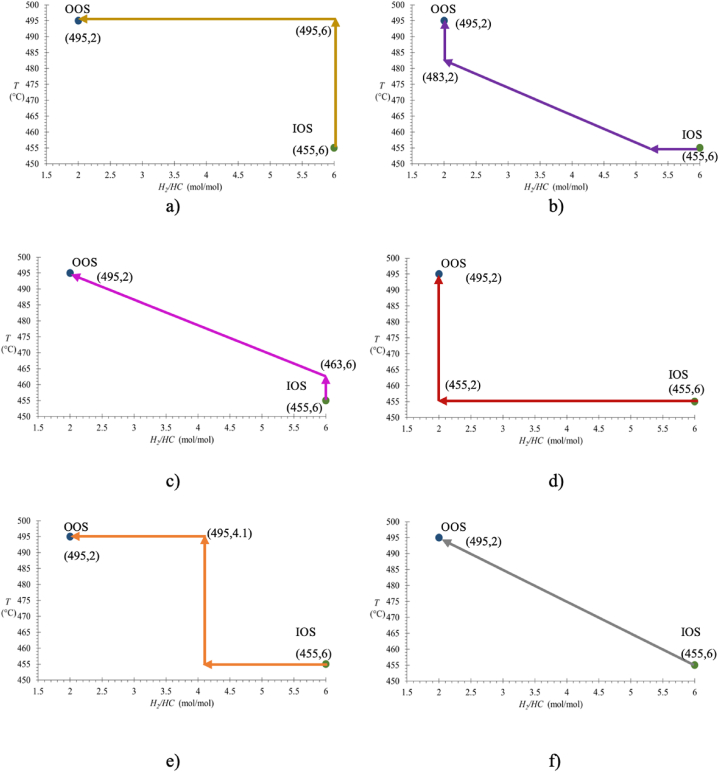
Transitional Operative Routes (TORs) for improving RON along with hydrogen production and quality: a) TOR 1, b) TOR 2, c) TOR 3, d) TOR 4, e) TOR 5, and f) TOR 6.

By keeping as an objective the final point of operative improvement for the operative transition based on looking for an increment in hydrogen production and its quality, it was identified and selected a set of different Transitional Operative Routes (TORs) that are shown in Fig. 6(a–f). Fig. 6a shows the TOR 1 starts with an increment in temperature from 450 °C up to 496 °C, followed by a decrement of the “*H*_*2*_*/HC*” ratio from 6 to 2. In Fig. 6b it is shown the TOR 2, as a first step “*H*_*2*_*/HC*” ratio was decreased from 6 to 5.5, followed by an increment in temperature from 450 °C to 477 °C with a simultaneous decrease of the “*H*_*2*_*/HC*” ratio from 5.5 to 2, ending with a final temperature increment from 477 °C to 496 °C. Fig. 6c shows the TOR 3 begins with an increment in temperature between 450 °C and 464 °C, followed by a decrease in the “*H*_*2*_*/HC*” ratio from 6 to 2, ending with a final increment in temperature from 464 °C to 496 °C. Fig. 6d shows the TOR 4 consisted in decreasing the “*H*_*2*_*/HC*” ratio from 6 to 2, followed by a final increment in temperature from 450 °C to 496 °C. In Fig. 6e it is shown the TOR 5, at the beginning it was analyzed a decrease in the “*H*_*2*_*/HC*” ratio of from 6 to 4, followed by a full increment in temperature from 450 °C to 496 °C, for ending with a decrease of the “*H*_*2*_*/HC*” ratio from 4 to 2. Finally, Fig. 6e shows the TOR 6 with a direct path from the IOS to the OOS with a simultaneous change of temperature with a direct increment from 455 to 495 °C along with a decrease of the “*H*_*2*_*/HC*” ratio from 6 to 2.

An analysis about the behavior of operative transitions was performed by comparing the average values that each dependent variable (*RON; %m of H*_*2*_; *(yH*_*2*_*)*_*R*_, and *(yH*_*2*_*)*_*S*_) took across the transitional route that was followed, along with the maximum achievable value for each dependent variable, estimated from the simultaneous changing of the 4 dependent variables, obtaining data shown in Table 4.

**Table 4:** Assessment of TORs regarding each response variable.

TOR No.	Average values estimated across each TOR				
	*RON (Position)*	%m of H_2_ (Position)	(yH_2_)_R_ (Position)	(yH_2_)_S_ (Position)	Average position
1	83.80 **(2)**	1.94 **(2)**	0.83 **(6)**	0.9531 **(5)**	**3.75**
2	81.02 **(5)**	1.88 **(3)**	0.86 **(2)**	0.9689 **(2)**	**3.00**
3	81.56 **(4)**	1.87 **(5)**	0.85 **(4)**	0.9642 **(4)**	**4.25**
4	80.31 **(6)**	1.86 **(6)**	0.87 **(1)**	0.9708 **(1)**	**3.50**
5	81.84 **(3)**	1.87 **(4)**	0.85 **(3)**	0.9642 **(3)**	**3.25**
6	84.24 **(1)**	2.16 **(1)**	0.84 **(5)**	0.9530 **(6)**	**3.25**
Objective Operative State (OOS) values	91.26	2.84	0.86	0.9607	

For the final assessment of TORs and selection of the one that could achieve the best conditions during the operative transitions, it was performed an analysis over the average results from each response variable, considering that routes with higher average values of each response variable, across the operative transition, allowed keeping the best productive quality during the operational change from the IOS until the OOS. A pros and cons analysis for each route was carried out and selection of routes, searching for the best transition, was as follows: route 1 was the 2nd best for *RON* values and (*%m of H*_*2*_), but the worst for the molar fraction of recycled hydrogen, and 5th for the molar fraction of produced hydrogen, afterwards, route 2 was the 5th one regarding the *RON* values, the 3rd one for the mass percentage of hydrogen produced (*%m of H*_*2*_), and the 2nd best for the rest of objective variables. Route 3 was the 4th one for RON and both, molar fraction of recycled and produced hydrogen, whilst the 5th one for the mass percentage of produced hydrogen (*%m of H*_*2*_), whereas route 4 generated the lowest *RON* values and of mass percentage of hydrogen production (*%m of H*_*2*_), with the highest molar fraction of both, recycled and produced hydrogen. Route 5 had the 4th position on the mass percentage of hydrogen production (*%m of H*_*2*_) and was the 3rd one in all the rest of objective variables. Finally, route 6 was the best in *RON* values and the mass percentage of hydrogen production (*%m of H*_*2*_), but the worst for the molar fraction of produced hydrogen and the 5th position for the molar fraction of recycled hydrogen. Therefore, considering the average position of each route, it was selected TOR No. 2 as the one with most advantages during transition. Nevertheless, this analysis can be used for assessing different operative options according to other industrial or productive priorities of the operative moments.

Additionally, routes that begin with increments in temperature can benefit the initial endothermic reactions of the CNR, which comprise cracking, cyclization, and isomerization; whereas further exothermic reactions are promoted with reductions in the *H*_*2*_*/HC* ratio, since hydrogen, as part of the reactants, could inhibit dehydrogenation reactions related to the formation of aromatic products that increase RON values. Routes Nos. 1 and 6 start with direct increments in temperature and that reflects the best RON values obtained. Routes 2, 4 and 5 start only with decrements of the *H*_*2*_*/HC* ratio at low temperatures, which benefits hydrogen production and quality not only for displacing kinetic equilibrium of dehydrogenation reactions, but also by allowing a hydrogen gradient concentration within the reactor that facilitates hydrogen diffusion and continuous production. Simultaneous changes increasing temperature and decreasing *H*_*2*_*/HC* ratio, as shown in routes 2, 3, and 6, seem to affect both endothermic and exothermic stages of the CNR reactions, but better results may be obtained starting with increments in temperature since the primary reaction stages are endothermic. Reductions in the *H*_*2*_*/HC* ratio can exhibit significant effects on reactions kinetics, once energetic stages of the CNR are initiated. Other criteria may be included for analyzing transitional operative routes, and this may result in different conclusions. It is also important considering lifetime of the catalyst and operative severity for avoiding chemical deactivation and coke formation on the catalyst, these constraints delimitate operative ranges of independent variables, not going below 2.0 for the *H*_*2*_*/HC* ratio and not above 510 °C in temperature. In this work, rather than suggesting absolute conclusions it is intended to provide an original method for considering a more detailed multi-objective optimization of the CNR, not only focusing on the optimum operative state, but also analyzing transitional routes in the process, which may help decision making during industrial operation.

### 3.2 Operative improvement

A search for optimum values of operative variables was performed by using *Design Expert* ® software. Results from numerical optimization were obtained for Improved Operative Conditions (IOC) with the following conditions for independent variables: *T* = 489.86 °C and *H*_*2*_*/HC* = 2.0; hence the IOS generated the following figures for the dependent objective variables *RON* = 90.72, (*%m of H*_*2*_) = 2.9 %, (*yH*_*2*_)_*R*_ = 0.8700 and (*yH*_*2*_)_*S*_ = 0.9653.

### 3.3 Pilot plant experiments

An experimental Pt-Re/Al_2_O_3_ catalyst was used for testing on a naphtha catalytic reforming semi-regenerative pilot plant. Firstly, a leaking test was carried out using nitrogen, at an initial stage, then followed by a hydrogen addition, both with a flowrate of 500 Nl/h, and at 700 kPa of pressure during 9 h. Furtherly a catalyst conditioning was performed for a period of 2 h, by setting an inlet flowrate of hydrogen of 83 Nl/h, under 7000 kPa of pressure, with 6000 kPa of pressure for recycled hydrogen, this procedure was completed after the leaking test, followed by an increased slope of temperature from 150 °C until 510 °C, with a rate of 36 °C/h.

Once the system was set up, we started the operative stage by opening a naphtha flowrate of 40 ml/min for 43 min, in order to set and stabilize the mass balance of naphtha flowing from the feed tank to the product tank, then naphtha flowrate was established to 30 ml/min, with a hydrogen flowrate of 500 Nl/h, under a pressure of 7000 kPa and changing temperatures from 400 °C, at day 1, 450 °C, at day 2, until 500 °C, at day 3.

#### 3.3.1 Characterization of naphtha feedstock and reformate products

Hydrotreated naphtha was fed to the pilot plant, this naphtha was previously analyzed by Multidimensional Chromatography, results were as follows, RON = 75.25, Density = 0.7518 g/ml and % (v/V) composition of: 47.78 % of naphthenes, 39.67 % of *i*-paraffins, 22.28 % of *n*-paraffins, 0.34 % of olefins, and 12.91 % of aromatic compounds.

For analyzing reformate products, 10 ml samples were taken from inlet and outlet of the pilot plant streamlines, these samples were analyzed by MC-GC and DHA-GC. Liquid samples from the pilot plant were taken at the beginning of the run (feedstock sample) and at the end of day 1 (reformate 1), and so on for day 2 (reformate 2) and day 3 (reformate 3). RON values obtained were 77.54, at the end of day 1, 84.03, at the end of day 2, and 92.25, at the end of day 3. It can also be observed on Table 5, that volumetric fractions of *n*-paraffins and naphthenes were decreasing during operational time, whereas the volumetric fraction of aromatic compounds was increased, which corresponds to the increments measured in RON values.

**Table 5:** Comparison between experimental data, obtained in pilot plant, with simulated data of RON, mol fraction of recirculated *H*_*2*_ and concentrations of paraffins, aromatics and naphthenes using operative conditions of a three days experimental run.

Day	Type of data	RON	Concentration [% (v/V)]			Mol fraction of recirculated *H*_*2*_
			n-Paraffins	Aromatics	Naphthenes	
1	*Simulated*	77.54	65.31	13.54	19.61	93.76
	*Experimental*	79.50	68.95	11.90	18.12	94.08
2	*Simulated*	84.03	56.20	24.30	15.92	89.92
	*Experimental*	85.93	60.48	21.03	17.54	89.55
3	*Simulated*	92.25	50.91	36.54	7.49	83.73
	*Experimental*	90.33	54.56	33.01	8.38	81.73
*Correlation coefficient*		0.9844	0.9987	0.9996	0.9693	0.9998

### 3.4 Comparative analysis of simulations, pilot plant results and industrial data

Table 5 shows a comparison between pilot plant experimental data and simulation results from changing conditions after 1, 2, and 3 days of process operation. This information was useful for correlating results from each type of data (simulated or experimental) either from RON values or naphtha composition, as well as the mol fraction of recirculated Hydrogen. An overall average correlation coefficient of 0.99036 was estimated.

Additionally, data from operative conditions of an industrial CNR plant were compared with pilot plant conditions and simulated results from an improved operative zone (see Table 6)

**Table 6:** Comparison of operative conditions at industrial scale, pilot plant, and simulation.

Type of variables	Industrial scale	Pilot plant scale	Optimum operative zone by simulation
*Operative*			
Temperature (°C)	442.99–498.74	430–500	482–500
*H*_*2*_*/HC* ratio (mol/mol)	2.00–5.16	2.00–4.00	2.00–3.50
*Response*			
Recirculated *H*_*2*_ (% mol)	79.0, 86.6 & 92.0	81.0–94.0	85.0–88.0
*RON*	83.2–92.8	77.0–92.0	87.0–94.0

**Fig. 7:**
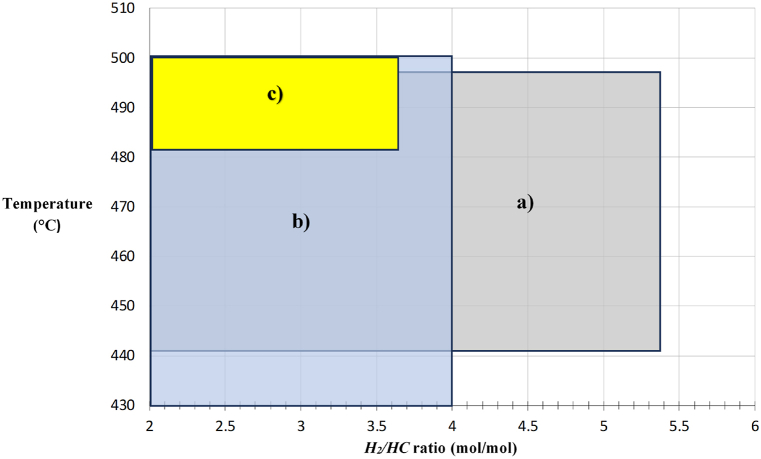
Diagram for identifying and intersecting operational zones: a) industrial scale; b) experimental pilot plant scale and c) optimal by simulation.

Fig. 7(a–c) show conditions for improved operative zones at pilot plant and industrial scales, observing that these zones match in certain ranges of operative conditions for a real operative zone. This is useful for identifying different operative points for each response variable, where it can be seen that both operative states (from the pilot plant and from industrial data) are coincident in a temperature value close to 500 °C, as the optimum reaction temperature of the process.

Fig. 7(a–c) show a limit temperature value of 500 °C and a lower limit value of the *H*_*2*_*/HC* ratio in 2.0, these values are considered as operative constraints for both independent variables, since there are effects on the catalyst, such like: coke formation, chemical deactivation, zeolite phase change, catalyst sintering and/or attrition. Temperature above 510 °C may cause an excess of coke formation and catalyst poisoning, hence best operative temperatures go around 498–503 °C, below this range catalytic reaction may occur with low efficiency and above the range catalyst poisoning will predominate. On the other hand, *H*_*2*_*/HC* ratio below 2.0 would decrease isomerization, cyclization and hydrocracking reactions, with higher coke formation rates resulting in a less efficient CNR process and a lower lifetime of the catalyst. *H*_*2*_*/HC* values above 6 would generate an excess of hydrogen displacing chemical equilibrium of aromatization and dehydrogenation reactions, which would reduce *RON* values. Therefore, any optimum operative point to be considered must be within constrained ranges of these two independent variables, in order to avoiding catalyst operative severity and a lower process profit.

**Fig. 8:**
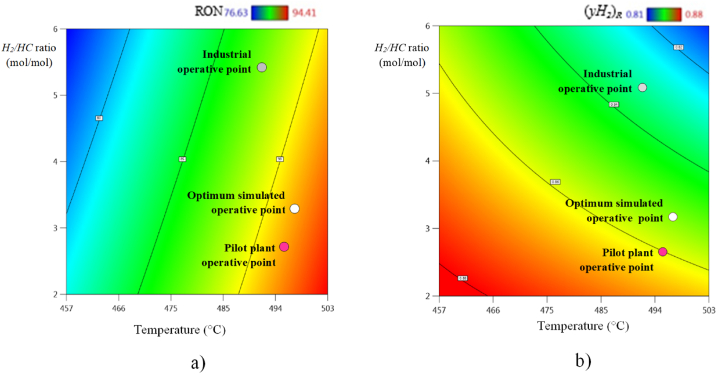
Multivariate surface response of the industrial, simulated, and pilot plant operative states for: a) octane number (*RON*) and b) molar fraction of recirculated hydrogen (*yH*_*2*_)_*R*_.

Fig. 8(aandb) show color gradient plots of Temperature vs. *H*_*2*_*/HC* molar ratio, and against *RON* values (see Fig. 8a) and molar fraction of recirculated hydrogen (*yH*_*2*_)_*R*_ (see Fig. 8b), on which three different operative points are identified, being: the Industrial Operative Point (IOP), the Pilot Plant Operative Point (PPOP), and the Optimum Simulated Operative Point (OSOP) These plots show the existing gaps between IOP and PPOP regarding the OSOP for both objective response variables. This demonstrates opportunity areas to be solved through an operative transition, in search of optimal conditions for increasing quality of both, the reformate and recycled hydrogen. By applying TORs analysis, it is feasible to optimize, not only the CNR process, but also the pathway to follow for an optimal transition minimizing any loss in productivity.

### 3.5 Error analysis and results comparison with literature

A previous study [30] was based on the development of an equations-oriented method for generating a mathematical model of a countercurrent CNR process. Multi-objective optimization was focused on aromatics yield and high-octane gasoline production, finding optimum values of operative variables as temperature and *H*_*2*_*/HC* molar ratio, including values for response variables regarding hydrogen production, as it can be seen in Table 7.

**Table 7:** Comparative analysis of results and error estimation regarding literature values.

Variable	Units	Optimization results		Relative estimated error (%)
		[30]	This work	
Temperature	°C	506.82	496.00	2.13
*H*_*2*_*/HC* ratio	mol/mol	2.05	2.00	2.44
(*yH*_*2*_)_R_	Mol fraction	0.924	0.870	5.84
(*yH*_*2*_)_S_	Mol fraction	0.945	0.965	2.11
RON	Dimensionless	98.14	90.72	7.56

Table 7 shows a maximum relative error value of 7.56 % for the RON value, whereas a minimum relative error value of 2.11 % for the mole fraction of produced hydrogen. These figures for a relative error are acceptable considering that optimization objectives may be different, but results are produced within feasible operative ranges.

## 4 Conclusions

A multivariate analysis was performed on simulation results and the surface response method was used for identifying an improved operative zone for the CNR process, it was achieved by involving independent variables like temperature (*T*) and the hydrogen/hydrocarbons ratio in the feedstock (*H*_*2*_*/HC*), in order to build quadratic multivariate models with interactions between variables, which generated response surfaces that were used for applying a surface superposition method for finding an optimum operative zone for improved values of objective variables, such as the highest *RON* of the reformate, with an increased production of hydrogen from the reactor, as well as the best quality of recirculated and produced hydrogen from the process, with all this information it was possible to establish an operative range of temperature between 457 °C and 503 °C, and an operative range for the hydrogen/hydrocarbon (*H*_*2*_*/HC)* ratio in the feedstock between 2.0 and 3.5 (mol/mol); henceforth generating a *RON* between 87 and 94, in the reformate, and a quality of recirculated hydrogen between 85 % and 88 %.

Transitional Operative Routes (TORs) were assessed and settled by considering an optimum objective state that resulted from quality improvement criteria, such as the search of an increment in reformate productivity and a higher production and quality of the recirculated and produced hydrogen. TOR No. 2 was selected since this allowed to maintain the highest quality and production of hydrogen, across the whole transition of operative conditions, keeping a *RON* value greater than the one obtained from TOR No. 4 and comparatively acceptable regarding TORs Nos. 3 and 5, only being slightly lower than TOR No. 1. For this, it is reasonable to conclude that, in this work it has been proposed a feasible approach for identifying the best route to carry out operative changes, in the search for improved operative conditions, while, simultaneously, it can be preserved profitability and productivity of the CNR process, whereas the quality of the reformate and hydrogen production are increased.

A numerical optimization procedure was performed by using *Design Expert* ® software keeping as the Improved Operative Conditions (IOC), the following ones: temperature (*T*) of 489.86 °C and hydrogen/hydrocarbon feed ratio (*H*_*2*_*/HC*) of 2.0 mol/mol, then these conditions produced an optimum estimated outcome, from each multivariate surface response model, of *RON* = 90.72; (*%m of H*_*2*_) = 2.90 %; (*yH*_*2*_)_*R*_ = 0.8700, and (*yH*_*2*_)_*S*_ = 0.9653.

Comparisons between results from simulation and pilot plant experiments showed an acceptable concordance for experimental runs under changing temperatures of 400 °C, 450 °C and 500 °C, for days 1, 2 and 3, respectively, maintaining a constant pressure of 7000 kPa, obtaining, for day 1, an experimental *RON* value of 79.54, when the estimated one by simulation was 79.70. For day 2, the experimentally measured *RON* value was 85.93 and the simulated *RON* value was 84.03. Finally, for day 3 the *RON* value measured from the reformate product of the pilot plant was 90.40, with a simulated *RON* value of 92.25, with these results it was estimated a correlation coefficient (*r*^*2*^) of 0.9844 between the whole set of experiments and estimations from the multivariate surface response model for *RON* values.

## CRediT authorship contribution statement

**F. Velázquez-Alonso:** Writing – original draft, Visualization, Validation, Software, Methodology, Investigation, Formal analysis, Data curation, Conceptualization. **C.A. González-Ramírez:** Writing – review & editing, Validation, Supervision, Resources, Methodology, Investigation, Funding acquisition, Formal analysis, Conceptualization. **J.R. Villagómez-Ibarra:** Supervision, Resources, Investigation. **E.M. Otazo-Sánchez:** Supervision, Resources, Investigation, Funding acquisition. **M. Hernández-Juárez:** Supervision, Resources. **F. Pérez-Villaseñor:** Supervision, Resources. **A. Castro-Agüero:** Supervision, Resources. **L.O. Alemán-Vázquez:** Validation, Supervision, Resources, Investigation, Data curation.

## Data availability statement

The original contributions presented in this study are included in the article. Further inquiries can be directed to the corresponding authors and data will be made available upon reasonable request.

## Declaration of competing interest

The authors declare the following financial interests/personal relationships which may be considered as potential competing interests: FABIOLA VELAZQUEZ ALONSO reports financial support was provided by 10.13039/501100013704National Council for Science and Technology (MEXICO). FABIOLA VELAZQUEZ ALONSO reports a relationship with 10.13039/501100013704National Council for Science and Technology (MEXICO) that includes: funding grants. If there are other authors, they declare that they have no known competing financial interests or personal relationships that could have appeared to influence the work reported in this paper.
